# An Unusual Cause of Gastrointestinal Bleeding in a Patient with Enteral Feeding

**DOI:** 10.5005/jp-journals-10018-1117

**Published:** 2014-07-28

**Authors:** Ömer Öztürk, Evrim Kahramanoglu Aksoy, Yagmur Can Dadakci, Ömer Basar

**Affiliations:** 1 Department of Gastroenterology, Hacettepe University, Ankara, Turkey; 2 Department of Anesthesiology and Reanimation, Hacettepe University, Ankara, Turkey

**Keywords:** Enteral feeding, Nasogastric feeding, Percutaneous gastrostomy.

## Abstract

**Abbreviations:** NG: Nasogastric; PEG: Percutaneous gastrostomy.

**How to cite this article:** Öztürk Ö, Aksoy EK, Dadakci YC, Basar Ö. An Unusual Cause of Gastrointestinal Bleeding in a Patient with Enteral Feeding. Euroasian J Hepato-Gastroenterol 2014;4(2):119.

To the Editor,

There are two main enteral feeding procedures: nasogastric (NG) tube feeding and percutaneous gastrostomy (PEG) which are used to improve the nutritional status of patients that are unable to meet their nutritional requirements through the oral route.^1^ In addition, these procedures have some complications.

A 89-year-old man who was being hospitalized in intensive care unit due to thromboembolic cerebrovascular disease underwent PEG tube insertion by conventional endoscopy because of dysphagia. Two days later, the patient had melena and bleeding from the PEG tube. At control endoscopy, a linear ulcer belonging to previous nasogastric tube which extended from cardia to the distal corpus of stomach was observed (Fig. 1).

For enteral nutrition, NG tube is usually used as an alternative to gastrostomy feeding. It can be placed easily and safely even in very sick patients with multiple comor-bidities by a minimally invasive procedure. Although it is suitable for short-term nutrition, the NG tubes are uncomfortable and agitated or confused patients easily displace them mistakenly or intentionally. The risks of aspiration, vomiting and local tissue trauma are the main complications of NG tube.^2^ Another safe and effective way for enteral nutrition, PEG tube insertion, also has complications, such as wound infection, leakage, hemorrhage, perforation, tube migration and buried bumper syndrome.^3,4^

This case presents an unexpected cause of bleeding due to NG tube, which was thought to be a result of PEG tube insertion. Endoscopists should keep the complications of NG tube in mind, before and after PEG tube insertion to minimize the complications.

**Fig. 1:**
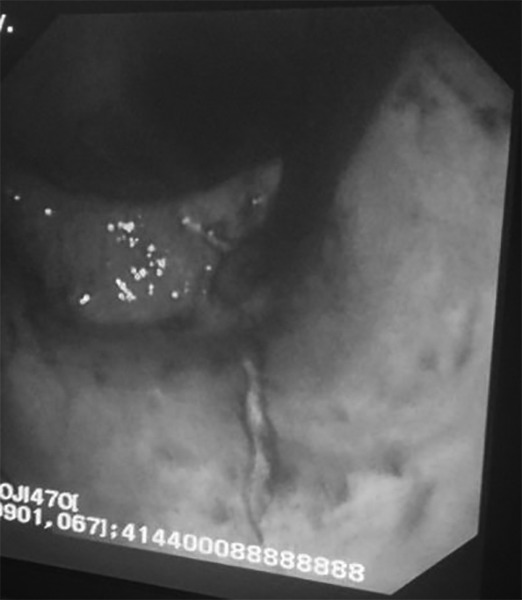
Endoscopic appearance of linear ulcer
